# Supplementary material for: Measuring the continuation of antidepressant exposure prior to, during, and after pregnancy: A scoping review protocol
Source: PLoS One. 2025 Oct 23;20(10):e0335144. doi: 10.1371/journal.pone.0335144 (PMC12548859; doi:10.1371/journal.pone.0335144)
Supplement: S1 File — (DOCX) [file pone.0335144.s001.docx]

**S1. Complete Literature Search Strategy**

| **Date** | December 12, 2024 |
| --- | --- |
| **Date limit(s)** | 2015-Current |
| **Language** | n/a |

| **Database [Platform]** *Searches run* | **Results** |
| --- | --- |
| Ovid MEDLINE(R) and Epub Ahead of Print, In-Process & Other Non-Indexed Citations and Daily 1946 to Dec 12, 2024 | 9047 |
| Ovid Embase Classic+Embase <1947 to 2024 Dec 16> | 4400 |
| Ovid EBM Reviews Cochrane Central Register of Clinical Trials – Dec 2024 | 8 |
| Ovid APA PsycInfo <1806 to Dec 2024> | 1717 |
| Clarivate Web of Science Core Collection (via U of T only) | 17353 |
| **TOTAL** | **32,525** |

***Medline Dec 12, 2024***

| **#** | **Searches** | **Results** |
| --- | --- | --- |
| 1 | *Antidepressive Agents/ | 31091 |
| 2 | "Selective Serotonin Reuptake Inhibitors"/ | 21670 |
| 3 | "Serotonin and Noradrenaline Reuptake Inhibitors"/ | 592 |
| 4 | Antidepressive Agents, Tricyclic/ | 10647 |
| 5 | *Monoamine Oxidase Inhibitors/ | 6386 |
| 6 | (anti*depress* or antidepress* or tricyclic* or TCA* or heterocyclic* or SNRI? or "monoamine oxidase inhibitor*" or MAOI* or SSRI? or "Selective Serotonin Reuptake Inhibitor?" or "Serotonin Uptake Inhibitor?" or "Serotonin Reuptake Inhibitor?" or “serotonin receptor” or psychotropic or "5-HT Uptake Inhibitor?" or "5-Hydroxytryptamine Uptake Inhibitor?" or "Serotonin and Norepinephrine Reuptake Inhibitor?" or "Serotonin and Noradrenaline Uptake Inhibitor?" or "SSRI? and NRI?" or "NRI? and SSRI?").tw,kf. | 173759 |
| 7 | (desipramine or imipramine or clomipramine or opipramol or trimipramine or lofepramine or dibenzepin or amitriptyline or nortriptyline or protriptyline or doxepin or iprindole or melitracen or butriptyline or dosulepin or amoxapine or dimetacrine or amineptine or maprotiline or quinupramine or zimeldine or fluoxetine or citalopram or paroxetine or sertraline or alaproclate or fluvoxamine or etoperidone or escitalopram or isocarboxazid or nialamide or phenelzine or tranylcypromine or iproniazide or iproclozide or moclobemide or toloxatone or oxitriptan or tryptophan or mianserin or nomifensine or trazodone or nefazodone or minaprine or bifemelane or viloxazine or oxaflozane or mirtazapine or bupropion or medifoxamine or tianeptine or pivagabine or venlafaxine or milnacipran or reboxetine or gepirone or duloxetine or agomelatine or desvenlafaxine or vilazodone).tw,kf. | 132900 |
| 8 | or/1-7 | 284416 |
| 9 | *Pregnancy/ or (pregnan* or parturition*).tw,kf. | 668524 |
| 10 | *lactation/ or *breast feeding/ or (lactat* or (milk secret* or express*) or (breastfeed* or Breast Fed or breastfed or wet nurs* or milk sharing*)).tw,kf. | 3582705 |
| 11 | *Postpartum Period/ or (postpartum* or puerperium).tw,kf. | 86822 |
| 12 | exp Infant, Newborn/ or Prenatal Care/ or Pregnant Women/ | 733481 |
| 13 | (prenatal* or perinatal* or antenatal* or periconception or preconception).tw,kf. or *Perinatal Care/ | 259545 |
| 14 | or/9-13 | 4842437 |
| 15 | ("36884236" or "26045370" or "35170756" or "38740587" or "31575566" or "32777011").ui. - key article PMIDs | 6 |
| 16 | 8 and 14 | 35976 |
| 17 | limit 16 to (yr="2015 -Current") | 18802 |
| 18 | 15 and 17 – validation only | 6 |
| 19 | 17 not ((Animals/ OR Animal Experimentation/ OR "Models, Animal"/ OR (animal* OR nonhuman OR non human OR Bovine or Cow? or Monkey$ OR rat OR rats OR mouse OR mice OR rabbit OR rabbit OR pig OR pigs OR porcine OR dog OR dogs OR hamster OR hamsters OR fish OR chicken OR chickens OR sheep OR cat OR cats OR raccoon OR raccoons OR rodent* OR horse OR horses OR racehorse OR racehorses OR beagle*).ti,ab.) ) | 9047 |

***Embase Classic+Embase <1947 to Dec 16 2024>***

| **#** | **Searches** | **Results** |
| --- | --- | --- |
| 1 | *Antidepressive Agent/ | 39062 |
| 2 | "Serotonin Uptake Inhibitor"/ | 55865 |
| 3 | "serotonin noradrenalin reuptake inhibitor"/ | 8806 |
| 4 | "tricyclic antidepressant agent"/ | 37121 |
| 5 | *"Monoamine Oxidase Inhibitor"/ | 7275 |
| 6 | (anti*depress* or antidepress* or tricyclic* or TCA* or heterocyclic* or SNRI? or "monoamine oxidase inhibitor*" or MAOI* or SSRI? or "Selective Serotonin Reuptake Inhibitor?" or "Serotonin Uptake Inhibitor?" or "Serotonin Reuptake Inhibitor?" or "5-HT Uptake Inhibitor?" or "5-Hydroxytryptamine Uptake Inhibitor?" or "Serotonin and Norepinephrine Reuptake Inhibitor?" or “serotonin receptor” or “psychotropic” or "Serotonin and Noradrenaline Uptake Inhibitor?" or "SSRI? and NRI?" or "NRI? and SSRI?").tw,kf. | 242884 |
| 7 | (desipramine or imipramine or clomipramine or opipramol or trimipramine or lofepramine or dibenzepin or amitriptyline or nortriptyline or protriptyline or doxepin or iprindole or melitracen or butriptyline or dosulepin or amoxapine or dimetacrine or amineptine or maprotiline or quinupramine or zimeldine or fluoxetine or citalopram or paroxetine or sertraline or alaproclate or fluvoxamine or etoperidone or escitalopram or isocarboxazid or nialamide or phenelzine or tranylcypromine or iproniazide or iproclozide or moclobemide or toloxatone or oxitriptan or tryptophan or mianserin or nomifensine or trazodone or nefazodone or minaprine or bifemelane or viloxazine or oxaflozane or mirtazapine or bupropion or medifoxamine or tianeptine or pivagabine or venlafaxine or milnacipran or reboxetine or gepirone or duloxetine or agomelatine or desvenlafaxine or vilazodone).tw,kf. | 175683 |
| 8 | or/1-7 | 414160 |
| 9 | *Pregnancy/ or (pregnan* or parturition*).tw,kf. | 949441 |
| 10 | *lactation/ or *breast feeding/ or (lactat* or (milk secret* or express*) or (breastfeed* or Breast Fed or breastfed or wet nurs* or milk sharing*)).tw,kf. | 4659205 |
| 11 | *Puerperium/ or (postpartum* or puerperium).tw,kf. | 116661 |
| 12 | exp Newborn/ or Prenatal Care/ or Pregnant Woman/ | 884320 |
| 13 | (prenatal* or perinatal* or antenatal* or periconception or preconception).tw,kf. or *Perinatal Care/ | 360412 |
| 14 | or/9-13 | 6216504 |
| 15 | 8 and 14 | 52042 |
| 16 | limit 15 to (yr="2015 -Current") | 26996 |
| 17 | limit 16 to (books or chapter or conference abstract or conference paper or "conference review" or data paper or editorial or erratum or letter or note or "preprint (unpublished, non-peer reviewed)" or short survey or tombstone) | 7890 |
| 18 | 16 not 17 | 19106 |
| 19 | 17 not ((Animals/ OR Animal Experimentation/ OR "Models, Animal"/ OR (animal* OR nonhuman OR non human OR Bovine or Cow? or Monkey$ OR rat OR rats OR mouse OR mice OR rabbit OR rabbit OR pig OR pigs OR porcine OR dog OR dogs OR hamster OR hamsters OR fish OR chicken OR chickens OR sheep OR cat OR cats OR raccoon OR raccoons OR rodent* OR horse OR horses OR racehorse OR racehorses OR beagle*).ti,ab.) ) | 4400 |

***Ovid EBM Reviews - Cochrane Central Register of Controlled Trials Dec 16 2024***

| **#** | **Searches** | **Results** |
| --- | --- | --- |
| 1 | Antidepressive Agents | 7850 |
| 2 | "Selective Serotonin Reuptake Inhibitors" | 4549 |
| 3 | "Serotonin and Noradrenaline Reuptake Inhibitors" | 130 |
| 4 | Antidepressive Agents, Tricyclic | 1486 |
| 5 | *Monoamine Oxidase Inhibitors | 854 |
| 6 | (anti*depress* or antidepress* or tricyclic* or TCA* or heterocyclic* or SNRI? or "monoamine oxidase inhibitor" or MAOI* or SSRI? “serotonin receptor” or “psychotropic” or "Selective Serotonin Reuptake Inhibitor" or "Serotonin Uptake Inhibitor" or "Serotonin Reuptake Inhibitor" or "5-HT Uptake Inhibitor" or "5-Hydroxytryptamine Uptake Inhibitor" or "Serotonin and Norepinephrine Reuptake Inhibitor" or "Serotonin and Noradrenaline Uptake Inhibitor" or "SSRI and NRI" or "NRI and SSRI").tw,kf. | 298 |
| 7 | (desipramine or imipramine or clomipramine or opipramol or trimipramine or lofepramine or dibenzepin or amitriptyline or nortriptyline or protriptyline or doxepin or iprindole or melitracen or butriptyline or dosulepin or amoxapine or dimetacrine or amineptine or maprotiline or quinupramine or zimeldine or fluoxetine or citalopram or paroxetine or sertraline or alaproclate or fluvoxamine or etoperidone or escitalopram or isocarboxazid or nialamide or phenelzine or tranylcypromine or iproniazide or iproclozide or moclobemide or toloxatone or oxitriptan or tryptophan or mianserin or nomifensine or trazodone or nefazodone or minaprine or bifemelane or viloxazine or oxaflozane or mirtazapine or bupropion or medifoxamine or tianeptine or pivagabine or venlafaxine or milnacipran or reboxetine or gepirone or duloxetine or agomelatine or desvenlafaxine or vilazodone).tw,kf. | 314 |
| 8 | #1 or #2 or #3 or #4 or #5 or #6 or #7 | 12157 |
| 9 | *Pregnancy or (pregnan* or parturition*).tw,kf. | 96122 |
| 10 | *lactation or *breast feeding or (lactat* or (milk secret* or express*) or (breastfeed* or Breast Fed or breastfed or wet nurs* or milk sharing*)).tw,kf. | 113042 |
| 11 | *Postpartum Period or (postpartum* or puerperium).tw,kf. | 17282 |
| 12 | exp Infant, Newborn or Prenatal Care or Pregnant Women | 30472 |
| 13 | (prenatal* or perinatal* or antenatal* or periconception or preconception).tw,kf. or *Perinatal Care | 5488 |
| 14 | #9 or #10 or #11 or #12 or #13 | 198928 |
| 15 | #8 and #14 | 1140 |
| 16 | limit 15 to yr="2015 -Current" | 876 |
| 17 | remove duplicates from 16 | 147 |
| 18 | #17 NOT (Animals OR Animal Experimentation OR (animal OR nonhuman OR non human OR Bovine or Cow or Monkey OR rat OR rats OR mouse OR mice OR rabbit OR rabbit OR pig OR pigs OR porcine OR dog OR dogs OR hamster OR hamsters OR fish OR chicken OR chickens OR sheep OR cat OR cats OR raccoon OR raccoons OR rodent* OR horse OR horses OR racehorse OR racehorses OR beagle)) | 8 |

***Ovid APA PsycInfo <1806 to Dec 2024 Week 3>***

| **#** | **Searches** | **Results** |
| --- | --- | --- |
| 1 | Antidepressant Drugs/ | 23141 |
| 2 | "Serotonin Reuptake Inhibitors"/ | 6050 |
| 3 | "Serotonin Norepinephrine Reuptake Inhibitors"/ | 525 |
| 4 | "Tricyclic Antidepressant Drugs"/ | 670 |
| 5 | "Monoamine Oxidase Inhibitors"/ | 1532 |
| 6 | (anti*depress* or antidepress* or tricyclic* or TCA* or heterocyclic* or SNRI? or "monoamine oxidase inhibitor*" or MAOI* or SSRI? or "Selective Serotonin Reuptake Inhibitor?" or "Serotonin Uptake Inhibitor?" or “serotonin receptor” or “psychotropic” or "Serotonin Reuptake Inhibitor?" or "5-HT Uptake Inhibitor?" or "5-Hydroxytryptamine Uptake Inhibitor?" or "Serotonin and Norepinephrine Reuptake Inhibitor?" or "Serotonin and Noradrenaline Uptake Inhibitor?" or "SSRI? and NRI?" or "NRI? and SSRI?").tw,hw. | 71319 |
| 7 | (desipramine or imipramine or clomipramine or opipramol or trimipramine or lofepramine or dibenzepin or amitriptyline or nortriptyline or protriptyline or doxepin or iprindole or melitracen or butriptyline or dosulepin or amoxapine or dimetacrine or amineptine or maprotiline or quinupramine or zimeldine or fluoxetine or citalopram or paroxetine or sertraline or alaproclate or fluvoxamine or etoperidone or escitalopram or isocarboxazid or nialamide or phenelzine or tranylcypromine or iproniazide or iproclozide or moclobemide or toloxatone or oxitriptan or tryptophan or mianserin or nomifensine or trazodone or nefazodone or minaprine or bifemelane or viloxazine or oxaflozane or mirtazapine or bupropion or medifoxamine or tianeptine or pivagabine or venlafaxine or milnacipran or reboxetine or gepirone or duloxetine or agomelatine or desvenlafaxine or vilazodone).tw,hw. | 37309 |
| 8 | or/1-7 | 87550 |
| 9 | Pregnancy/ or (pregnan* or parturition*).tw,hw. | 63608 |
| 10 | *lactation/ or *breast feeding/ or (lactat* or (milk secret* or express*) or (breastfeed* or Breast Fed or breastfed or wet nurs* or milk sharing*)).tw,hw. | 322720 |
| 11 | *Postnatal Period/ or (postpartum* or puerperium).tw,hw. | 20626 |
| 12 | Prenatal Care/ or Expectant Mothers/ | 3573 |
| 13 | (prenatal* or perinatal* or antenatal* or periconception or preconception).tw,hw. or *Perinatal Period/ | 44551 |
| 14 | or/9-13 | 406799 |
| 15 | 8 and 14 | 8329 |
| 16 | limit 15 to (yr="2015 -Current") | 3549 |
| 17 | remove duplicates from 16 | 3542 |
| 18 | 17 not ((Animals/ OR Animal Experimentation/ OR "Models, Animal"/ OR (animal* OR nonhuman OR non human OR Bovine or Cow? or Monkey$ OR rat OR rats OR mouse OR mice OR rabbit OR rabbit OR pig OR pigs OR porcine OR dog OR dogs OR hamster OR hamsters OR fish OR chicken OR chickens OR sheep OR cat OR cats OR raccoon OR raccoons OR rodent* OR horse OR horses OR racehorse OR racehorses OR beagle*).ti,ab.) ) | 1717 |

***Clarivate Web of Science Core Collection (via U of T only) Dec 16, 2024***

(TS=(prenatal* OR perinatal* OR antenatal* OR periconception OR postpartum* OR puerperium OR lactat* OR "milk secret*" OR express* OR breastfeed* OR "Breast Fed" OR breastfed OR "wet nurs*" OR "milk sharing*" OR pregnan* OR parturition*))

AND

(TS=(desipramine OR imipramine OR clomipramine OR opipramol OR trimipramine OR lofepramine OR dibenzepin OR amitriptyline OR nortriptyline OR protriptyline OR doxepin OR iprindole OR melitracen OR butriptyline OR dosulepin OR amoxapine OR dimetacrine OR amineptine OR maprotiline OR quinupramine OR zimeldine OR fluoxetine OR citalopram OR paroxetine OR sertraline OR alaproclate OR fluvoxamine OR etoperidone OR escitalopram OR isocarboxazid OR nialamide OR phenelzine OR tranylcypromine OR iproniazide OR iproclozide OR moclobemide OR toloxatone OR oxitriptan OR tryptophan OR mianserin OR nomifensine OR trazodone OR nefazodone OR minaprine OR bifemelane OR viloxazine OR oxaflozane OR mirtazapine OR bupropion OR medifoxamine OR tianeptine OR pivagabine OR venlafaxine OR milnacipran OR reboxetine OR gepirone OR duloxetine OR agomelatine OR desvenlafaxine OR vilazodone OR antidepress* OR SNRI* OR "monoamine oxidase inhibitor*" OR MAOI* OR SSRI* OR "Selective Serotonin Reuptake Inhibitor*" OR "Serotonin Uptake Inhibitor*" OR "5-HT Uptake Inhibitor*" OR "Serotonin and Norepinephrine Reuptake Inhibitor*" OR psychotropic))

NOT

(TS=(animal* AND (model* OR mouse OR mice)))

Years: 01/01/2015-12/16/2024

Number of results: 17353

**Grey Literature Search Strategy:**

***Google Scholar:***

- The first fifty article search results from Google Scholar (searched on Sept 4, 2024)
- Search strategy: “antidepressant” AND “pregnancy”

***Grey Matters***

Databases in Grey Matters by Canada’s Drug Agency (formerly CADTH) were searched using keywords designed to mirror the primary search strategy. Initial searches using broad terms such as “antidepressant” yielded records (e.g., clinical guidelines, surveillance data). However, when additional terms such as “pregnancy,” “prenatal,” “perinatal,” or “maternal” were added, no relevant grey literature sources were identified. We therefore manually reviewed each listed data source for relevance to pregnancy-related antidepressant use, searching within the listed resources. Despite this effort, no eligible grey literature records were identified for inclusion using the Grey Matters resource.
